# A hybrid type III effectiveness–implementation study designed to test implementation best practices of deploying a *Screen-Triage-Treat* approach to cervical cancer screening and management utilizing self-collected HPV DNA testing in Chokwe District, Mozambique

**DOI:** 10.1186/s12889-025-25730-5

**Published:** 2025-11-25

**Authors:** Troy D. Moon, Mila P. Salcedo, Elzier Mavume-Mangunyane, Mohsin Sidat, Cesaltina Lorenzoni, Ellen Baker, Gustavo Amorim, Valerie A. Paz-Soldán, Kathryn T. Kampa, Kathleen Condon, Harriett Myers, Jahit Sacarlal

**Affiliations:** 1 Department of Tropical Medicine and Infectious Diseases, Celia Scott Weatherhead School of Public Health and Tropical Medicine, Tulane University, New Orleans, LA USA; 2 Department of Gynecologic Oncology and Reproductive Medicine, The University of Texas MD Anderson Cancer Center, Houston, TX USA; 3 Faculty of Medicine, University Eduardo Mondlane, Maputo, Mozambique; 4 National Cancer Control Program, Ministry of Health, Maputo, Mozambique; 5 Maputo Central Hospital, Maputo, Mozambique; 6 Department of Biostatistics, Vanderbilt University Medical Center, Nashville, TN USA

**Keywords:** Human papilloma virus, Cervical cancer, Self-collection, Protocol, Implementation science, HIV, Mozambique

## Abstract

**Background:**

The current World Health Organization (WHO) recommendation for cervical cancer screening utilizing HPV DNA testing is now being endorsed by the Mozambican Ministry of Health (MOH). Initial studies showing the feasibility of screening in the Mozambican context have been encouraging, with women participants reporting a preference for self-collection approaches. A high HPV prevalence among women living with HIV/AIDS will result in a significantly higher number of women requiring follow up, many of whom can receive thermal ablation at the primary level to avoid creating bottlenecks for those who require specialized follow up. Studies designed to evaluate best practices for implementation among a high-risk, lower-resourced population within Mozambique´s already busy HIV care and treatment services are needed to provide the evidence required national scale-up this approach.

**Methods:**

The overall aim of this study is to evaluate implementation best practices for deploying a *Screen-Triage-Treat* approach to cervical cancer screening and management utilizing self-collected HPV DNA testing among HIV-infected women in care within select health facilities of Chókwè District, Mozambique. The main objectives are 1) to develop an in-depth understanding of the HPV *Screen-Triage-Treat* care cascade within HIV care and treatment based on a collaborative, exploration-focused process with local stakeholders; 2) to evaluate the effectiveness of the *Screen-Triage-Treat* approach utilizing self-collected HPV DNA testing; and 3) to use the Consolidated Framework for Implementation Research (CFIR) to identify factors that explain site- and provider-level variation in the implementation of the *Screen-Triage-Treat* approach to cervical cancer screening and management.

**Discussion:**

This study will contribute to the literature related to cervical cancer screening and management, and to implementation science by providing information on how screening and management can be implemented within the high-volume HIV care and treatment clinics of Mozambique. Studies that generate evidence on barriers and facilitators to the uptake of this approach, and that identify context-specific implementation strategies are important for large-scale adoption. The knowledge gained from this study will be used to assist decision makers in determining a course of action for increasing cervical cancer screening and management coverage, optimizing the intervention’s impact, and translating our findings into evidence-based programming.

**Trial registration:**

NCT06810739 (Protocol ID: 2024–1641). Registered 04 February 2025, at https://clinicaltrials.gov/

**Supplementary Information:**

The online version contains supplementary material available at 10.1186/s12889-025-25730-5.

## Background

Every year approximately 300,000 women die from cervical cancer, with over 90% of those deaths occurring in low-and middle-income countries (LMICs) [1–3]. The majority of cervical cancers are preventable through vaccination against high-risk types of human papillomavirus (HPV), the virus that causes nearly all cervical cancers, and/or by screening for and treatment of precancerous lesions among women already infected with HPV. While deaths from cervical cancer have declined dramatically in high-income and some middle-income countries, cervical cancer mortality has continued to increase in developing countries. At current trajectories, cervical cancer is projected to cause an estimated 443,000 deaths globally in 2030 [2]. In May 2018, the World Health Organization (WHO) announced a global call to action towards the elimination of cervical cancer, underscoring renewed political will to make elimination a reality [4].

Cervical cancer, classified as an AIDS-defining illness, is the most frequently detected cancer among women living with HIV (WLWH) worldwide [5, 6]. Studies have shown that the risk of cervical cancer is up to sixfold higher among HIV-infected women compared to HIV-uninfected women [7]. However, in spite of widespread access to antiretroviral therapy (ART) in LMIC, the incidence and mortality associated with cervical cancer among WLWH persists, likely due to a continued lack of resources and infrastructure for high-quality cervical cancer screening, diagnosis and treatment [8, 9].

The dramatic decline in cervical cancer deaths seen in higher-income countries has been attributed, in part, to increased availability of HPV testing and/or cervical cytology, which enable precancerous lesions to be detected and treated before they become cancer. Today, visual inspection with acetic acid (VIA) is a screening method used in many LMIC. VIA is cheaper and easier to implement than cervical cytology and allows women to receive immediate treatment with cryotherapy or thermal ablation after a positive screen, which can help avoid treatment delays and loss to follow-up. However, challenges include limited screening coverage of the population, lack of public education regarding cervical cancer screening, and few health care providers trained to properly diagnose and treat precancerous lesions. In addition, scaling up VIA has proven difficult due to high inter-provider variability, leading to lower sensitivity to detect abnormalities. Molecular testing to detect high-risk HPV, responsible for roughly 95% of cervical cancer cases, has the potential to increase the quality and reliability of cervical cancer screening services [10].

In 2021, the WHO recommended HPV testing among HIV-infected women aged 25–49 years for all cervical cancer screening programs worldwide [11]. Despite this recommendation, access to affordable HPV testing remains a barrier, and to date, there is limited field experience with HPV testing across sub-Saharan Africa (SSA). HPV self-collection holds great promise in improving access to screening and promoting equity by increasing control over where, when, and how women are screened. HPV self-collection has been shown to be highly acceptable among both women and providers. Since the sample can be taken by the woman to be screened, this procedure eliminates the need for a pelvic exam in the vast majority of women who test HPV negative, thereby reducing costs and opportunity costs associated with multiple visits. Women who test positive will need additional evaluation. A recent meta-analysis indicates that the clinical performance of HPV testing to detect cervical pre-cancer or cancer is equivalent between self-collected and provider-collected specimens [10].

Mozambique has one of the highest burdens of cervical cancer in the world, with an age-standardized incidence rate of 50.2 and mortality rate of 38.7 per 100,000 women [12, 13]. Cervical cancer ranks as the most frequent cancer among women in Mozambique, with an estimated 5,300 new cases and 3,800 related deaths reported each year [14]. The prevalence of HIV in women ages 15–49 in Mozambique was estimated at 15.4% in 2021 [15]. Cervical cancer screening was introduced in the country in 2009 using VIA followed by immediate treatment with ablation (cryotherapy or thermal ablation) or referral for LEEP if the cervical lesion(s) did not meet the criteria for ablation [13, 14, 16]. One challenge is that many of the women who screen positive with VIA, are not undergoing treatment with ablation at the same visit. As a result, many women who screen positive are subsequently lost to follow-up and never receive treatment. Most women with cervical cancer in Mozambique therefore present with advanced stage disease and the majority die of their disease.

The current WHO recommendation for cervical cancer screening utilizing primary HPV testing is now being endorsed by the Mozambican Ministry of Health (MOH) [17]. Initial studies showing the feasibility of this approach in the Mozambican context have been encouraging, with women participants reporting a preference for self-sampling approaches [18]. However, these studies to date were conducted amongst eligible women accessing family planning services in higher-resource areas near the nation´s capital of Maputo. Studies designed to evaluate best practices for implementation among a high-risk, lower-resourced population within Mozambique´s already busy HIV care and treatment services are needed to provide the evidence-base required to take this approach to scale.

We summarize below our protocol for a hybrid type III effectiveness-implementation study of HPV self-collection within the HIV care and treatment program in Chókwè District, Mozambique, describing the conceptual frameworks that have guided its development, the different phases that will be employed throughout its implementation, and the measures we propose for evaluating its implementation and effectiveness.

## Methods/design

The introduction of a new intervention into a clinical setting is a complex process and requires support and buy-in at each facility. We will use a hybrid type III effectiveness-implementation design, with the primary focus on the testing of an implementation strategy, while simultaneously continuing to observe and gather information on the effectiveness of a clinical intervention, under real world conditions, for which effectiveness data has already largely been determined.

This protocol was developed following the SPIRIT guidelines (Standard Protocol Items: Recommendations for Intervention Trials) [19]. The review protocol was pre-registered at https://clinicaltrials.gov/.

### Aims/objectives

The overall aim of this study is to evaluate implementation best practices for deploying a *Screen-Triage-Treat* approach to cervical cancer screening utilizing self-collected HPV DNA testing among HIV-infected women in care within select health facilities in Chókwè District, Mozambique. The main objectives of this study are:

1. To develop an in-depth understanding of the HPV *Screen-Triage-Treat* care cascade within the HIV care and treatment services based on a collaborative, exploration-focused process with local stakeholders.
2. To evaluate the effectiveness of the *Screen-Triage-Treat* approach to cervical cancer screening utilizing self-collected HPV DNA testing.
3. To use the Consolidated Framework for Implementation Research (CFIR) to identify factors that explain site-level and provider-level variation in the implementation of the *Screen-Triage-Treat* approach to cervical cancer screening utilizing self-collected HPV DNA testing.

Table 1 shows the SPIRIT diagram including the schedule of enrollment, intervention, and assessment.

**Table 1:** SPIRIT diagram: schedule of enrollment, intervention, and assessment

Study Period	Pre-Implementation Phase	Implementation Phase	Follow-up Phase	Post-implementation Evaluation
Timepoint	Month −2 to 0	Month 0–30	Month 1–30	Month 31–33
Enrollment:				
Site preparation and staff training	X			
Two-week pilot assessment period	X			
Model participant enrollment (1–2/day)		X		
Participant eligibility screening		X		
Informed Consent		X		
Intervention:				
Self-collected HPV DNA testing (Screen)		X		
HPV result reporting (same-day CHC; weekly in periphery)		X		
VIA and thermal ablation if eligible (Triage-and-treat)		X		
Referral to higher level of care for LEEP/colposcopy in not eligible for ablation		X		
Assessments:				
Baseline retrospective data (past 12 months)	X			
Process mapping/group model building workshop and qualitative interviews (Objective 1a-c)	X	X		
Quantitative data collection (HPV, VIA, treatment rates)		X		
Implementation Outcomes (adoption, fidelity, reach, acceptability)		X	X	
Qualitative provider interviews (CFIR domain)			X	
Statistical analysis of primary outcome (proportion of HPV + women treated, Objective 2)			X	
Analysis of secondary outcomes (implementation outcomes, Objective 3)			X	
Dissemination workshops and final reporting				X

^*^CHC Carmelo Hospital of Chokwe, *HPV* Human papilloma virus, VIA Visual inspection with acetic acid, *CFIR* Consolidated Framework for Implementation Research

### Study setting

**Fig. 1:**
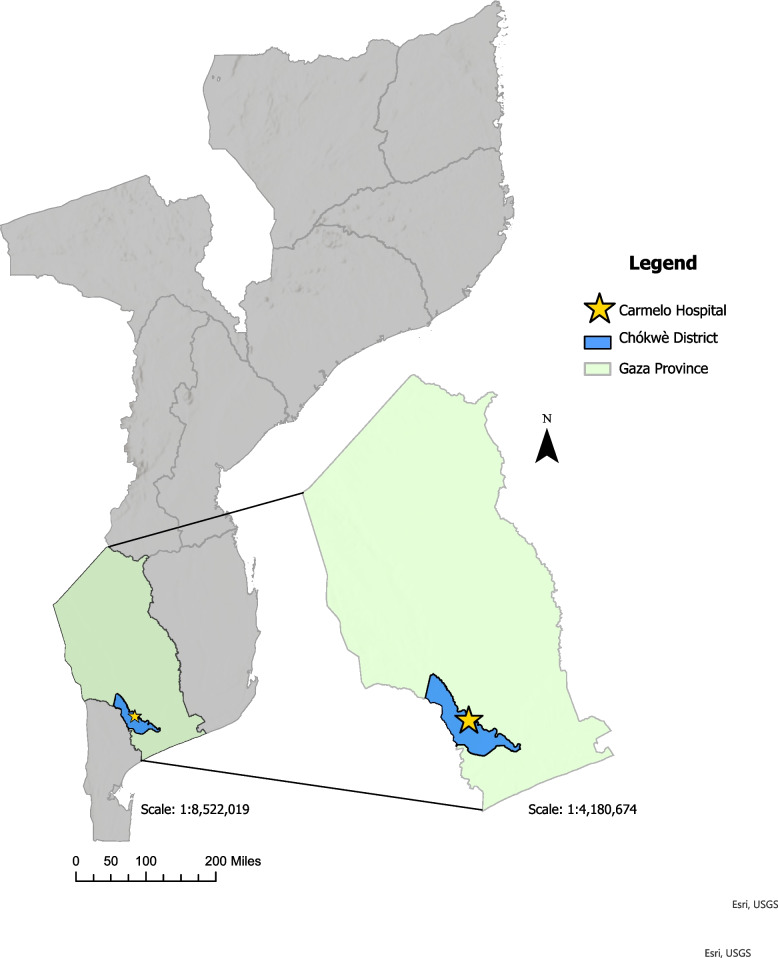
Map of Mozambique with Chókwè District in Gaza Province highlighted. *Map generated by Kathrine Gruber

This study will take place at Carmelo Hospital of Chókwè (CHC), as well as three peripheral health facilities (*Centro de Saúde (CS) da Cidade; CS Urbano; and CS de Chalucuane*) within the district network of facilities that respond to CHC. CHC is a reference hospital for HIV and TB in the district of Chókwè, a large district in Gaza Province in southern Mozambique (Fig. 1). Chókwè District is predominantly rural, with a surrounding catchment population of approximately 200,000 inhabitants. Agriculture is the predominant source of local income for roughly 60% of the population, while approximately 40% of the population migrates seasonally to South Africa, seeking work in the mines, a recognized driver of the district’s high HIV and TB burden [20]. Per the latest national HIV prevalence study in 2021, Gaza Province had the highest provincial HIV prevalence in the country at 20.9% [15].

These facilities were selected due to their 1) high HIV prevalence catchment area; 2) established HIV care and treatment services; 3) on-site expertise in use of the GeneXpert platform; 4) capacity on-site for cervical pre-cancer treatment with thermal ablation; and 5) capacity for referral for colposcopy and/or LEEP at the Hospital Rural of Chókwè.

**Fig. 2:**
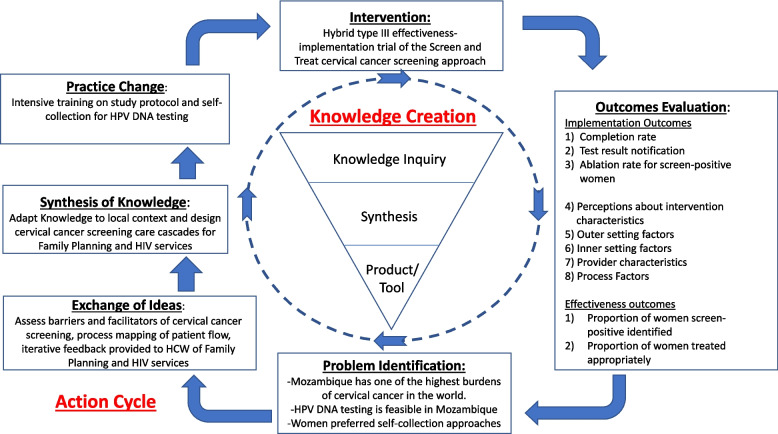
Knowledge-to-Action (KTA) conceptual framework

This study is guided by the Knowledge-to-Action (KTA) conceptual framework (Fig. 2). The KTA Framework is comprised of two interrelated components: Knowledge Creation and an Action Cycle. Each component is further comprised of multiple phases which can employ iterative processes and can overlap [21, 22]. Implementation science calls for systematic and flexible implementation models which facilitate the adaptation of evidence-based interventions that are then subsequently deployed and sustained [23]. These models ideally consider institutional context so that the intervention is easily integrated within the local setting and thus ensures a higher likelihood of implementation success. The KTA Framework provides a schematic to help guide, monitor, and evaluate the translation of knowledge into actionable steps for improvement.

### Objective 1

To develop an in-depth understanding of the HPV *Screen-Triage-Treat* care cascade within the HIV care and treatment services based on a collaborative, exploration-focused process with local stakeholders.

Hypothesis: Adaptations in the cervical cancer care cascade that takes into consideration local, real-world inputs, will result in greater uptake in screening and subsequent treatment amongst HPV screen positive women seen in the HIV care and treatment services.

#### Specific Objective 1a

To explore potential barriers and facilitators to current facility-based cervical cancer screening strategies through in-depth interviews with health care providers.

For this sub-objective, we will employ qualitative, semi-structured key informant interviews of health workers and clinicians with knowledge of the HIV care and treatment services.

##### Theoretical framework for qualitative interviews

A semi-structured interview guide will be developed based on the Theoretical Domains Framework (TDF) [24, 25]. The TDF is based on psychological theory and is used to identify determinants of individual behavior [24]. The TDF integrates psychological theories for behavior change across 84 constructs that are organized into 14 domains. TDF domains include *knowledge; skills; social/professional role and identify; beliefs and capabilities; optimism; beliefs about consequences; reinforcement; intentions; goals; memory, attention and decision processes; environmental context and resources; and social influences *[24]. TDF was selected because it offers a theoretical way to understand implementation processes, which can then be mapped to the TDF domain components.

##### Study participants and sampling

We will employ purposive sampling of select administrators and health workers who are directly involved in the care of women accessing HIV care and treatment services at CHC and the selected peripheral health facilities. This could include physicians, nurses, and/or clinical officers. We will conduct 10–15 interviews, ensuring at least one physician interview, one superior nurse interview, and a mixed representation from the remaining participants. This includes both supervisory staff and general clinical staff, as well as one to two clinicians from each of the selected peripheral health facilities.

##### Data collection

Semi-structured interviews will be conducted face-to-face in a quiet and private location and audio recorded. The interview guide will include a combination of questions specific to the TDF domains and which elicits a deeper understanding of the barriers and facilitators to the full realization of current cervical cancer screening strategies.

##### Analysis plan for qualitative interviews

Immediately following each interview, the audio files will be uploaded and stored in an electronic database: *Research Electronic Data Capture (REDCap) system*. REDCap is a secure, web-based application designed to support a variety of data capture options for research studies [26]. The audio files will be transcribed verbatim and subsequently coded, aided by the TDF framework, by two researchers. Using NVivo, we will pre-populate all TDF codes and guidelines to facilitate coder inter-reliability. We will categorize and organize the data as an initial thematic analysis, allowing us to identify recurrent themes and explore interview responses. After each analyst has coded five interviews, they will assess their inter-coder reliability, discussing disagreements in efforts to achieve consensus. If consensus cannot be achieved, the study’s three principal investigators (PI) will engage to arbitrate disputes.

#### Specific Objective 1b

To conduct a process mapping exercise that will inform the design of a cervical cancer *Screen-Triage-Treat* care cascade within the HIV care and treatment services.

For this sub-objective, the PIs will spend one-week with the local study clinicians to conduct prospective observations of the flow of a woman through the HIV care and treatment services, from the point of initial nurse triage and then to any other services they access throughout their time at the facility that day, including clinician visit, psychosocial care visit, pharmacy, and laboratory. We will attempt to observe 10–15 women moving through the HIV services and 2–3 women at each of the peripheral health facilities.

##### Data collection

A mapping guide will be developed based on health facility standard operating procedures (SOPs) and first-hand observation of patient flow through the HIV care and treatment services with documentation consisting of 1) what is happening at each step; 2) where it takes place; 3) who is involved; 4) how quick patients move between steps; and 5) inflection points where patient management decisions around HPV self-screening could be made.

##### Analysis plan for mapping exercises

A process map is a diagram of the major action steps and decision inflection points within a given setting [27]. Collected data will be translated into visual process maps, using standardized symbols and notations to represent various steps, decision points, and interactions within the workflow. In-depth analysis will be conducted to identify bottlenecks, redundancies, and areas for improvement. Key performance indicators will be established to measure the efficiency and effectiveness of the processes. Finally, recommendations for process enhancements will be formulated, with an emphasis on optimizing workflows, streamlining activities, and ultimately improving overall organizational performance.

#### Specific Objective 1c

To conduct a group model-building workshop for sharing, testing, and revising strategies for the *Screen-Triage-Treat* care cascade implementation.

Prior to study launch we will conduct a series of group model-building exercises with local administrators and clinicians familiar with CHC´s HIV care and treatment services, as well as community leaders. This workshop will sensitize attendees to the aims and methods of the study as well as engage them in a group exercise for sharing, testing, and revising systems maps of the new cervical cancer *Screen-Triage-Treat* care cascade, in order to define potential problem areas of implementation. Attendees will have the opportunity to ask questions and make recommendations to the studies roll-out plans. This workshop will enable attendees to find leverage for change and participate in a collaborative, exploration-focused approach to define a shared vision for implementation.

##### Expected outcomes of the group model-building workshop

The group model-building workshop will consist of group discussions facilitated for the purpose of reviewing and documenting identified barriers and facilitators to cervical cancer screening in Mozambique and geared towards eliciting attendee feedback as to the potential areas and strategies for improvement. The team will jointly review the key informant interview results and process mapping results to better understand “what actually is occurring during each step”, versus “what they would like to occur, or thinks is occurring”. Attendees will undergo scenario analysis exercises in which they will examine potential alternative implementation options, ideally arriving at a shared vision for future implementation. Expected deliverables from this workshop include 1) stakeholder designed implementation plans; 2) a plan for ongoing monitoring and evaluation of screening implementation, experienced challenges, and identified potential solutions; and 3) development of dissemination plans of study results upon study completion.

### Specific objective 2

To evaluate the effectiveness of the *Screen-Triage-Treat* approach to cervical cancer screening utilizing self-collected HPV DNA testing.

Hypothesis: Utilization of the *Screen-Triage-Treat* approach will result in a significant number of women with precancerous cervical lesions being identified and successfully treated.

#### Two week assessment period

Prior to study enrollment launch, we will conduct a two-week pilot assessment period during which we will evaluate and modify the specific systems and processes of the HPV self-sampling care cascade. During this period, enrollment will be limited to 1–2 participants per day. Each day, study staff will review the day´s activities and the movement of the participants through the system, soliciting feedback for potential changes. All procedures will be followed, but participants will not contribute to study data.

#### Model participant period

At the conclusion of the two-week assessment period, we will begin enrollment of “model” participants. During this time, participants will be enrolled, contribute data, and complete longitudinal follow-up, but enrollment will still be limited to 1–2 participants per day. Data will be presented to the study PIs daily, documenting the number of women accepting self-screening; the number of women who were HPV screen positive; and the number of eligible women who received thermal ablation on the same day (or were referred for higher level care if needed). Once the site has enrolled a minimum of 20 participants (5 each at each of the peripheral health facilities) and the study staff feel comfortable with the enrollment procedures, the site will go live.

#### Study participants and enrollment period

Women accessing health care for HIV care and treatment services at CHC, between the ages of 25–49 years of age will be approached for enrollment into the study and subsequent cervical cancer screening. Following the “Model” Participant period, enrollment of participants will continue for a total of 30 months (Table 2).

**Table 2:** Inclusion and Exclusion criteria

Inclusion Criteria	Exclusion Criteria
Women 25–49 years, accessing HIV care and treatment services	Physical or mental impairment that inhibits participation in the study
Not being pregnant	Pregnant women or < 6 weeks post-partum
Patients with a cervix (women who have undergone a total hysterectomy with removal of the cervix are not eligible)	

#### Study procedures and the cervical cancer *Screen-Triage-Treat *care cascade

After providing informed consent, the study clinician will open a Case Report Form (CRF) for each patient which starts with a brief questionnaire which will include the participant’s demographic information and medical history, including HIV viral load and CD4 counts, previous cervical cancer screening results if previously screened, and result of rapid pregnancy test performed that day. Each participant will then be accompanied to a private location where the study clinician will provide counseling and instruction on how to appropriately perform the self-collection vaginal swab technique. Data on HPV testing, VIA results, and referrals along each step of the process will be documented in the CRF.

#### Laboratory procedures at CHC

Directly following collection of the vaginal swab sample, the participant will be taken back to the waiting area and her sample will be taken to the laboratory for HPV testing using the Xpert HPV Test on the GeneXpert platform (Cepheid, Sunnyvale, CA, USA). This point-of-care test takes approximately one hour to get results. Participants will be asked if they agree with having any remaining sample volume after HPV testing used for future research. If they agree, a future use research informed consent document will be reviewed and signed.

#### Laboratory procedures at CS da Cidade, CS Urbano, and CS de Chalucuane

Directly following collection of the vaginal self-swab sample, the sample will be placed into a vial with PreserveCyt (ThinPrep System, Hologic Inc, Marlborough, Massachusetts, USA). At a minimum of once per week, samples will be transported to CHC and subsequently tested using the XPert HPV test as described above. Participants will be asked if they agree with having any remaining sample volume after HPV testing used for future research. If they agree, a future use research informed consent document will be reviewed and signed. Results will be picked up the following week when new samples are brought into CHC and distributed to each health facility.

#### Visual Assessment for Treatment (VAT)

**Fig. 3:**
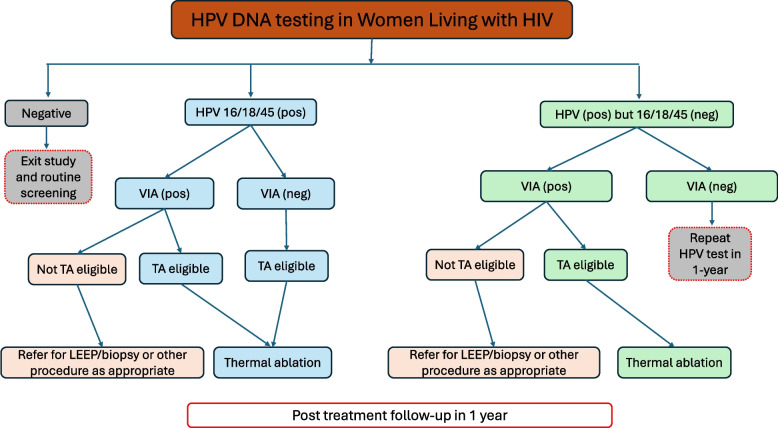
Flow diagram for HPV DNA testing in Women Living with HIV. *HPV = Human papillomavirus; VIA = Visual Inspection with Acetic Acid; TA = Thermal ablation; and LEEP = Loop Electrosurgical Excision Procedure

Women with a positive HPV test will undergo visual assessment for treatment (VAT) followed by ablation using thermal ablation, if eligible (Fig. 3). Women with lesions not eligible for ablation will be referred to the neighboring Hospital Rural of Chókwè for LEEP or other treatment, as appropriate. During the examination, the health care provider will also use a smart phone device to take images/videos of the cervix so that he or she will be able to document the test results and perform quality control. Participants will be asked if they agree with having their images/videos used for future research. If they agree, a future use research informed consent document for the photo will be reviewed and signed. Images/videos collected to be used for future research will be de-identified.

Following assessment as described above, women who screen positive will be eligible for ablative therapy if there is no suspicion of invasive or glandular disease, and if:

- The transformation zone (TZ) is fully visible, the whole lesion is visible, and it does not extend into the endocervix; or
- The lesion is type 1 TZ (when it only contains ectocervical components); or
- The lesion is type 2 TZ where the probe tip will achieve complete ablation of the squamocolumnar junction (SCJ) epithelium, i.e., where it can reach the upper limit of the TZ. Sometimes the SCJ can be seen high in the canal, but a probe tip would not reach it.

Women are not eligible for ablative therapy if there is any suspicion of invasive or glandular disease (i.e., adenocarcinoma or adenocarcinoma in situ), and:

- The TZ is not fully visible because it is endocervical (Type 3 TZ), or
- It is a type 2 TZ where the SCJ is out of reach of the probe tip.
- The lesion(s) cover > 75% of the cervix.

If the above criteria for ablation are not met, the woman will be referred to a gynecologist at the closest facility (Hospital Rural of Chókwè). The clinician will evaluate the patient with colposcopy, cervical biopsy, endocervical curettage, and/or immediate LEEP as appropriate per standard guidelines. Women diagnosed with invasive cervical cancer or requiring further diagnostic workup not feasible in this facility will be referred to either Xai-Xai Provincial Hospital (HPX) or Maputo Central Hospital (HCM).

#### Date collection

Prior to intervention roll-out, baseline data will be retrospectively collected for the prior 12 months at each of the selected study sites, including the number of women accessing care for HIV care and treatment services each month; the number of women screened for cervical cancer through standard of care protocols; the proportion of screen-positive women who then received treatment or referral for treatment, and the length of time between screening positive and receiving treatment, if appropriate. Data will be collected by study team members (physician, nurse, or medical technicians) utilizing a paper-based instrument and then uploaded into a password-protected, tablet-based, online research data capture (REDCap) database. This method allows for the recording of demographic information, medical and medication history, and any pertinent laboratory test results. Data quality control will be conducted by study investigators who will review all completed paper-based study instruments and confirm the accuracy of data entered into REDCap.

#### Outcome measures

Our primary outcome measure is the number of women screened and the proportion of screen-positive women successfully undergoing treatment. Secondary outcomes measures will include other implementation outcomes (see Specific Objective 3 below), and if successful, will be utilized to inform future research to take this approach to scale across Mozambique.

#### Sample size and power calculations

**Fig. 4:**
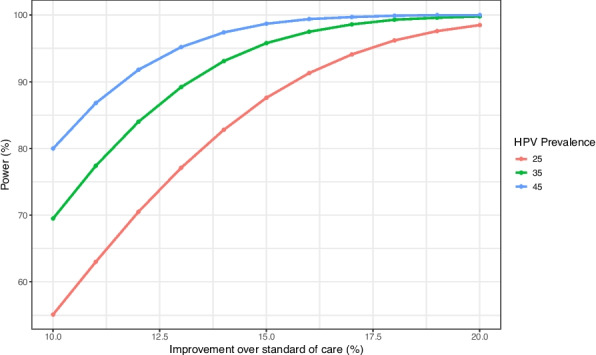
Power calculations

We used our primary outcome, more specifically, the proportion of HPV screen positive women that will successfully undergo treatment, to power the study. We used preliminary data to estimate a sample size sufficient to detect, with 90% power, an improvement of at least 15% in the proportion of women undergoing treatment compared to the current standard of practice. CHC currently sees approximately 1,200 HIV adult outpatient visits per month, of which 52% are female (*n* = 624) and approximately 75% of women are between the ages of 25–49 years (*n* = 468). Of these, we conservatively expect approximately 60% will not have been screened in the last three years and meet screening eligibility (*n* = 280). Based on preliminary data, the prevalence of HPV-positivity among WLWH was estimated to be approximately 40%; however, in our calculations, we again took a more conservative approach and assumed an HPV prevalence of 35% (*n* = 98). Under the current standard of care scenario we estimate that approximately 36% of HPV screen positive women go on to receive treatment (CHC currently performs, on average, 35 thermal ablation procedures per month). Figure 4 below shows statistical power curves to detect improvements, through utilization of the HPV self-testing *Screen-Triage-Treat* approach, varying from 10 to 20% in the proportion of HPV-screen positive women undergoing same day thermal ablation, compared to standard of care. We used a type-I error fixed at 5%, two-sample, two-sided binomial test, and assumed trimestral cumulative data to plot the curves, for different prevalences of HPV. Calculations were performed using the R software (*pwr* R package) [28]. Based on Fig. 4, we will be able to detect over 15% improvements in the proportion of HPV-screen positive women undergoing same day thermal ablation compared to standard of care, while assuming a prevalence of 35% or even lower at 25%, with at least 90% power.

Thus, by screening at least 280 HIV-infected women per month for a total of 30 months (total *n* = 8,400, of which 35% are expected to be HPV-screen positive), we expect to have at least 441 (15% of 2,940) added HPV-screen positive women who will subsequently undergo treatment, compared to standard of care alone. Of the 8,400 women screened, we anticipate that roughly 6,900 will be screened at CHC and roughly 500 will come from each of the three peripheral health facilities.

#### Statistical analysis plan

Using data captured during implementation of the *Screen-Triage-Treat* approach for cervical cancer screening, we will evaluate intervention fidelity as a function of 1) *Completion Rate*: the proportion of enrolled women who turned in a self-collected sample for HPV DNA testing; 2) *Test Result Notification Rate*: the proportion of women who were notified of their HPV DNA test result on the same day as sample collection; and 3) *Treatment Rate*: the proportion of screen-positive women who were treated with thermal ablation, if appropriate, or referral for higher level of care.

Patients’ characteristics and clinical values will be summarized in tables and figures. Continuous variables will be presented as medians and interquartile ranges. Categorical variables as frequencies and percentages. Descriptive statistics will be done for the overall population and stratified intervention status and study site. Continuous variables will be compared via the Mann–Whitney-Wilcoxon test, and categorical variables will be compared via the chi-square test. We will use a generalized linear model (GLM) with the logit link to and perform an interrupted time series to assess the impact of the intervention and the proportion of patients who subsequently underwent treatment for cervical cancer. The model will regress the outcome on intervention status (binary variables, yes/no), calendar time (in months), and an interaction between the two terms. By doing this we will be able to assess the immediate impact of the intervention (also known as level change) as well as its effect over time (sustained effect of the intervention) compared to the counterfactual scenario of no intervention being implemented. Analysis and comparisons will be performed overall, combining all sites, as well as being stratified by health facilities. We will adjust for relevant demographic and clinical characteristics, such as age, CD4 count, and viral load, among other factors. Continuous variables will be modeled via restricted cubic splines. We will again use GLM with a gaussian link to estimate the impact of the intervention on the number of WLWH diagnosed with HPV, adjusting for the same covariates as above, and following the same steps as above (e.g., another interrupted time series). If the normality assumption is violated, we plan to use ordinal regression models as an alternative. We do not expect issues with statistical over-fitting.

### Specific objective 3

To use the Consolidated Framework for Implementation Research (CFIR) to identify factors that explain site-level and provider-level variation in the implementation of the *Screen-Triage-Treat* approach to cervical cancer screening utilizing self-collected HPV DNA testing.

Hypothesis: Quantitative and qualitative interviewing with health facility providers will generate important information about intervention uptake and fidelity that will inform subsequent implementation scale-up.

The introduction of a new intervention into a clinical setting is a complex process and requires support and buy-in at each facility. As a hybrid type III effectiveness—implementation study, this proposal is predominantly focused on testing the implementation strategy of our intervention. However, we will also observe and gather information on the clinical intervention's impact on relevant outcomes.

**Fig. 5:**
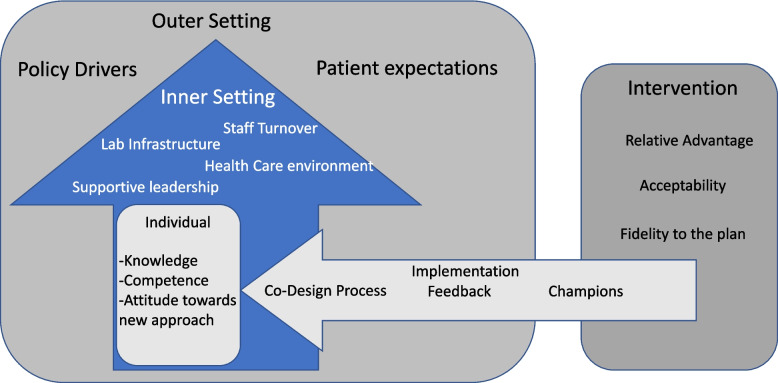
Consolidated framework for implementation research: factors hypothesized to impact implementation

We will conduct a mixed-methods evaluation, employing qualitative, semi-structured interviews and a quantitative survey questionnaire to explore provider experiences with implementing the *Screen-Triage-Treat* approach for cervical cancer screening. We will evaluate constructs from the Consolidated Framework for Implementation Research (CFIR), including perceptions about the *Intervention Characteristics* (e.g., relative advantage of the approach); *Outer Setting factors* influencing use (e.g., perception of patient needs and resources); *Inner Setting factors* impacting uptake and fidelity to the intervention (e.g., compatibility, relative priority, available resources); *Characteristics of Individuals* (e.g., knowledge and beliefs about the intervention); and *Process Factors* (e.g., champions, planning activities) within each health facility. We will look at how these factors influenced uptake and fidelity throughout implementation (Fig. 5).

#### Study participants and sampling

Similar to strategies used for Specific Objective 1a, we will employ purposive sampling to select health workers who are directly involved in the recruitment and care of women enrolled in the study under Specific Objective 2. We will conduct 10–15 key informant interviews, ensuring at least one physician interview and a mixed representation from the remaining supervisory and general clinical staff. Due to staff turnover, participants may or may not be the same health workers interviewed under Specific Objective 1a.

#### Data collection

Semi-structured interviews will be conducted face-to-face in a quiet and private location and audio recorded. The CFIR framework will be employed to develop an interview guide to generate a robust description of the social, facility culture, personnel factors, and intervention characteristics that influenced successes and/or challenges with implementation. These measures elicit a multi-level emic perspective of the implementation process and suggestions for future improvements. We will assess the degree of implementation uptake (*adoption*) and fidelity (*the degree to which the intervention is delivered as intended*) at each site. Questions designed to elicit data about the intervention’s appropriateness will include provider perceptions of the clinical relevance of the intervention; ease of incorporation into routine workflow; and perception of the value of its continued use.

#### Analysis plan for qualitative interview

Immediately following each interview, the audio files will be uploaded into a REDCap database. The audio files will be transcribed verbatim and coded by two researchers. Analysts will be blinded to the level of implementation adoption and fidelity to avoid bias. Using NVivo, we will prepopulate all CFIR codes and guidelines to facilitate coder inter-reliability. We will categorize and organize the data as an initial thematic analysis, allowing us to identify recurrent themes and explore interview responses. After each analyst has coded five interviews, they will assess their inter-coder reliability, discussing disagreements in efforts to achieve consensus. If consensus cannot be achieved, PIs Moon, Sacarlal, and Salcedo will engage to arbitrate disputes. Our data interpretation objective is to identify constructs that distinguish facilities and/or providers with high and low implementation success, which will provide us information about the constructs that influence implementation and inform future strategy development as we scale up the intervention.

#### Dissemination plans

Evidence generated from this study will be used to inform national and global efforts to scale-up effective and efficient screening and treatment programs.

A team comprising the PIs, co-researchers and technical staff of the HIV/AIDS program in Chókwè District will guide the analysis, interpretation and subsequent submission and publication of results. Upon completion of the primary data and analysis before finalizing a final report, the results will be shared in small communities made up of health professionals, community forums, and other stakeholders where the study is being conducted to confirm and contextualize results. Also, the data will be used to inform and involve public authorities and civil society organizations for taking necessary measures for the prevention, diagnosis and treatment of cervical cancer. In addition to the national dissemination of results in the form of a final report, the program will produce several abstracts and publications for international dissemination describing relevant findings to our primary and secondary objectives.

## Discussion

This study will evaluate the effectiveness and implementation of a new cervical cancer screening and management program that utilizes primary HPV self-collection as part of a *Screen-Triage-and-Treat* approach. Our focus will be among women accessing the HIV care and treatment services of select hospitals/health facilities in Chókwè District, Mozambique. The hybrid type III effectiveness implementation design will allow us to test the continuum of care for this new HPV-based *Screen-Triage-and-Treat* approach, while we simultaneously gather information on the intervention’s impact on outcomes under real world conditions. Findings from this study could be scaled-up to additional regional health facilities of Chókwè District and eventually nationwide where the physical and human resource conditions for implementing a robust cervical cancer screening program exists.

This study will contribute to the literature related to cervical cancer screening and management, and to implementation science by providing evidence on how HPV-based *Screen-Triage-and-Treat* can be implemented within the high-volume HIV care and treatment clinics of Mozambique. Studies that generate evidence on barriers and facilitators to these approaches, and that identify context-specific implementation strategies are important for large-scale adoption. The knowledge gained from this study will be used to assist decision makers in determining a course of action for increasing cervical cancer screening and management coverage, optimizing the intervention’s impact, and translating our findings into evidence-based programming.

### Limitations

Despite the strengths of this hybrid type III effectiveness-implementation study, several limitations should be acknowledged. First, as an implementation-focused study, effectiveness outcomes may be influenced by contextual factors, such as variations in healthcare infrastructure, workforce capacity, and patient characteristics across study sites. These differences may limit the generalizability of our findings to other settings with different resource availability or policy environments. Second, implementation strategies will be adapted to local contexts, which, while enhancing real-world applicability, introduces heterogeneity in intervention delivery. This variability may make it challenging to determine which specific components contributed most to implementation success or failure. Third, the study relies on both qualitative and quantitative data collection methods, including surveys, interviews, and routine health system data. While this mixed-methods approach strengthens our understanding of implementation outcomes, self-reported data may be subject to recall bias, social desirability bias, or incomplete reporting. Fourth, given the hybrid design, effectiveness outcomes may be assessed in a non-randomized manner, limiting causal inference. Confounding variables, such as differences in patient populations or preexisting healthcare initiatives, may influence observed outcomes. Fifth, sustainability and scalability remain concerns, as the study duration may not capture long-term adoption and maintenance of the intervention. External factors such as policy changes, funding availability, or workforce turnover may impact the continued success of implementation strategies beyond the study period.

## Supplementary Information

Supplementary Material 1.

## Data Availability

Quantitative datasets generated and/or analyzed during the current study will be made available at [https://osf.io/t2hrw/] Qualitative data can be made available upon reasonable request to the corresponding author and subject to approval by the ethics committee overseeing the study.

## Abbreviations

ART: Antiretroviral therapy
CFIR: Consolidated framework for implementation research
CHC: Carmelo Hospital of Chókwè
CIBS: Comité Interinstitucional de Bioética para a Saúde
CRF: Case report form
CS: Centro de Saúde
CNBS: Comité National de Bioética para Saúde
DNA: Deoxyribonucleic acid
FAMED: Faculdade de Medicina (Faculty of Medicine)
GLM: Generalized linear model
HCM: Hospital Central de Maputo
HIV: Human immunodeficiency virus
HPV: Human papillomavirus
HPX: Hospital Provincial de Xai-Xai
IRB: Institutional review board
KTA: Knowledge to action
LEEP: Loop electrosurgical excision procedure
LMIC: Low- and middle-income country
MOH: Ministry of health
NAS: National academy of sciences
NCCP: National cancer control program
PEER: Partnerships for enhanced engagement in research
REDCap: Research electronic data capture
SCJ: Squamocolumnar junction
SSA: Sub-Saharan Africa
TDF: Theoretic domains framework
TZ: Transformation zone
UEM: University Eduardo Mondlane
USAID: U.S. Agency for international development
VAT: Visual assessment for treatment
VIA: Visual inspection with acetic acid
WHO: World Health Organization
WLWH: Women Living with HIV
